# Targeting microRNAs in diabetic retinopathy: from pathogenic mechanisms to therapeutic potentials

**DOI:** 10.3389/fendo.2025.1664604

**Published:** 2025-09-23

**Authors:** Jiajun Chen, Jingjing Zhang, Changlei Li, Ling Wang, Long Tao, Shasha Xue, Fenglei Wang

**Affiliations:** ^1^ Department of Ophthalmology, The Affiliated Hospital of Qingdao University, Qingdao, China; ^2^ Department of Hepatobiliary and Pancreatic Surgery, The Affiliated Hospital of Qingdao University, Qingdao, China

**Keywords:** diabetic retinopathy, miRNA, oxidative stress, biomarkers, therapeutic targets

## Abstract

Diabetic retinopathy (DR), a prevalent microvascular complication affecting diabetic patients, imposes a significant global burden. Current therapies like anti-vascular endothelial growth factor (anti-VEGF) agents, offering limited efficacy in early stages and posing challenges related to invasiveness and recurrence. This underscores the urgent need for novel strategies targeting early intervention. This review proposes a unifying hypothesis: microRNAs (miRNAs) function as master regulators that integrate and amplify hyperglycemia-induced damage across multiple pathological axes—oxidative stress, inflammation, neurodegeneration, and vascular dysfunction. Dysregulation of specific miRNAs not only contribute to DR pathogenesis through multi-target modulation of key pathways but also exhibit stage-specific expression patterns in biofluids, positioning them as promising non-invasive biomarkers. Furthermore, miRNA-based therapeutic interventions, leveraging tools like quantitative reverse transcriptase PCR (qRT-PCR), droplet-based digital PCR (ddPCR), and microarrays for profiling, hold revolutionary potential to modulate key pathological cascades, and ultimately enable precision management strategies for early intervention and prevention of DR progression.

## 1 Introduction

Diabetes mellitus (DM) is a chronic metabolic disorder pathologically defined by persistent hyperglycemia resulting from peripheral insulin resistance (hallmark of type 2 diabetes mellitus, T2DM) or insulin deficiency (characteristic of type 1 diabetes mellitus, T1DM). According to the International Diabetes Federation Diabetes Atlas (10th Edition), DM affected 10.5% of the global population aged 20–79 years (536.6 million individuals) in the baseline year, with epidemiological modeling projecting an alarming 16.2% relative increase to 12.2% (783.2 million cases) by 2045 (1). Diabetic retinopathy (DR), the most prevalent microvascular complication of DM, affects approximately 90% of T1DM patients and 60% of T2DM patients within 20 years of diagnosis (2).

Hyperglycemia impairs the integrity of retinal microvasculature and induces pathological angiogenesis (3). As a consequence of these vascular pathologies, the progression of DR unfolds in a stepwise manner. Initially, non - proliferative DR (NPDR), characterized by the presence of microaneurysms and intraretinal hemorrhages, gradually evolves into proliferative DR (PDR), marked by the emergence of neovascularization (4). Despite current therapies such as anti-vascular endothelial growth factor (anti-VEGF) therapy offering clinical benefits, their invasive nature and adverse effects—including vitreous hemorrhage, endophthalmitis, and retinal detachment from repeated injections (5)—highlight the pressing need to vigorously explore therapeutic strategies that either intervene at the early stage of DR or effectively prevent its development.

MicroRNAs (miRNAs), a class of small non-coding RNAs, are evolutionarily conserved post-transcriptional regulators that fine-tune gene expression by binding to target mRNAs, leading to translational repression or degradation (6). These molecules are integral to diverse biological processes such as cellular growth, apoptosis, fibrosis, and senescence, and their dysregulation has been implicated in numerous diseases, including cancer, diabetes, and cardiovascular disorders (7, 8). Since the landmark discovery of miRNA involvement in chronic lymphocytic leukemia in 2002, research has expanded to uncover their roles in complex ocular diseases, particularly DR (9). Recent studies highlight miRNAs as pivotal mediators of DR pathogenesis, influencing critical mechanisms like angiogenesis, inflammation, oxidative stress, and neurodegeneration. This review synthesizes current research on miRNA-mediated regulatory networks in DR, evaluates their diagnostic and therapeutic applicability.

## 2 Global epidemiology of DR

Global epidemiological modeling projects DR to be a persistent global public health crisis. As of 2020, an estimated 130 million individuals worldwide were afflicted with DR. Moreover, it is forecasted that by 2045, the number of patients developing DR will be near 160 million (10). Vision-threatening complications, including vision-threatening diabetic retinopathy (VTDR) and clinically significant macular edema (CSME), collectively impair over 47 million individuals, with VTDR affecting 6.17% (28.54 million; 95% CI: 25.12–32.34 million) and CSME 4.07% (18.83 million; 95% CI: 3.42–4.82%) of the global diabetic population (10).

As the fifth leading cause of blindness among working-age adults (50 years of age and older) (11). DR exhibits striking geographical disparities: Asia shoulders the highest global burden, propelled by rapid urbanization, dietary transformations, and a burgeoning population of individuals with diabetes (12, 13). In China, with its large diabetic population and aging demographic, approximately 19.5 million people with diabetes are affected by DR. Among them, one - fifth have reached the VTDR stage (14). India demonstrates stark regional contrasts, with DR prevalence ranging from 12.27% in central regions to 34.06% in northern states, alongside an urban-rural divide (17.4% vs. 14.0%) (15). In contrast to the escalating DR burden in low- and middle-income regions, high-income nations paradoxically confront persistent DR challenges despite their comprehensive screening programs and widespread access to anti-VEGF therapies. In the United States, where 37.6 million adults were diagnosed with diabetes in 2021, epidemiological modeling revealed that 9.60 million individuals (95% UI: 7.90–11.55 million) — representing 26.43% (95% UI: 21.95–31.60) of the diabetic population — were affected by DR. Alarmingly, 1.84 million patients (95% UI: 1.41–2.40 million) progressed to VTDR, translating to a 5.06% prevalence rate (95% UI: 3.90–6.57) among diabetics (16). In Europe, DR is observed in 25.7% of individuals diagnosed with type 1 or 2 diabetes. Among this population, 18.5% exhibit mild to moderate NPDR, while 3.7% develop DME. Despite a steady decline in DR prevalence across Japan over the past decade, the prevalence rate remains alarmingly high — affecting 23.5% of Japanese diabetes patients (17).

## 3 Updated diagnosis and therapy of DR

ETDRS classification remains the gold standard for DR due to robust validation predicting progression, but its complexity impedes clinical adoption. This led to the ICDR Severity Scale— a streamlined adaptation of the ETDRS framework—which now serves as the most commonly adopted benchmark in clinical workflows due to its simplified five-tier staging (18, 19). Modern diagnostics integrate multimodal imaging: ultra-widefield (UWF) retinal imaging captures peripheral lesions, and optical coherence tomography angiography (OCTA) visualizes microvasculature (20, 21). AI enables automated lesion detection and predictive modeling for scalable screening (22, 23). These innovations enhance staging accuracy, facilitate progression monitoring, and optimize therapeutic decisions.

Anti-VEGF therapy (ranibizumab, bevacizumab, aflibercept) is the established first-line treatment for center-involved DME and a validated option for PDR, with extensive evidence demonstrating efficacy in edema reduction and vision improvement (24, 25). However, the DRCR.net Protocol W trial indicated that while proactive anti-VEGF treatment for NPDR prevents progression to PDR or DME, it does not yield superior long-term visual outcomes compared to initial observation with as-needed treatment upon complication development (26). Furthermore, the apparent improvement in DR severity with anti-VEGF often masks persistent underlying retinal ischemia, and lesions frequently recur rapidly after therapy cessation (24). For PDR, panretinal photocoagulation (PRP) remains the standard, effectively reducing vision loss risk (27). Emerging therapies focus on enhancing efficacy and durability while reducing injection frequency. Faricimab, a bispecific antibody inhibiting both VEGF and angiopoietin-2 (Ang-2)/Tie pathways, achieved visual gains comparable to aflibercept in DME but with superior anatomic outcomes and significantly extended dosing intervals (≥12–16 weeks for >50-70% of eyes at 1 year), addressing vascular instability more effectively (28–30). Nevertheless, treatment selection must weigh adherence challenges, injection burden, and patient preference against PRP’s durability.

Recent research focuses on identifying novel biomarkers – defined as biological molecules indicating physiological or pathological processes – to enable early detection of DR, halt progression, and prognostic outcomes (e.g., predicting NPDR→PDR transition), thereby guiding resource allocation and treatment strategies (31). Multiple clinical studies have identified 12-HETE and 2-piperidone in plasma/serum as potential DR biomarkers, which exhibited superior diagnostic performance to HbA1c in DR assessment using multiplatform metabolomics approaches (32). Circulating miRNAs represent a revolutionary frontier. Emerging evidence demonstrates that dynamic alterations in miRNA expression profiles in biofluids (e.g., serum, aqueous humor) strongly correlate with specific DR progression stages. These miRNAs exhibit highly sensitive and specific differential expression patterns, enabling non-invasive liquid biopsies to distinguish NPDR from PDR (33). This molecular stratification provides a crucial complement to structural imaging modalities, offering profound potential not only for earlier and more precise diagnosis but also for unveiling novel therapeutic targets to address the underlying pathophysiology of DR.

## 4 miRNAs biogenesis and silencing mechanisms

**Figure 1:**
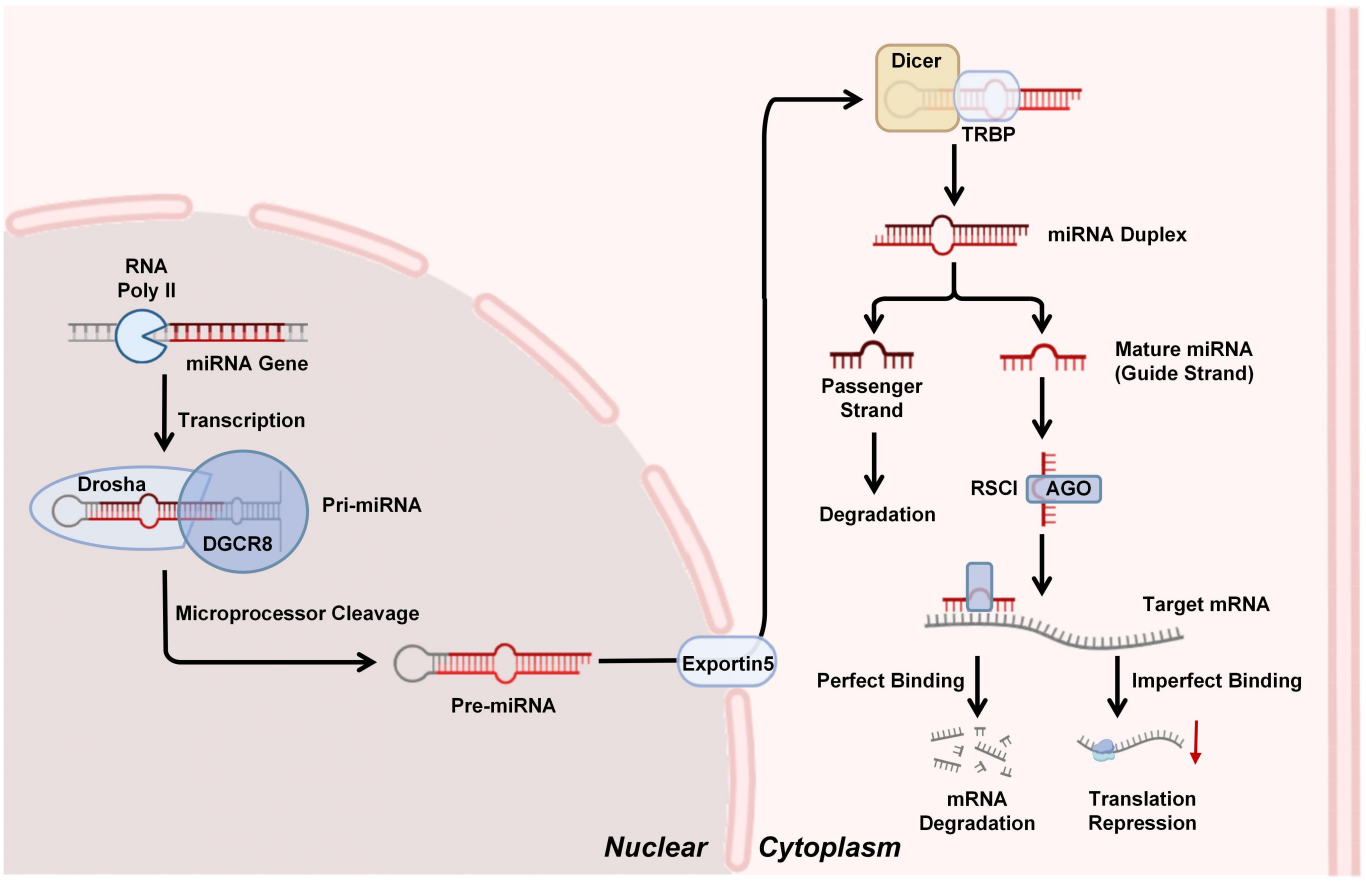
miRNAs biogenesis and silencing mechanism: miRNA genes are transcribed by RNA polymerase II (Pol II) into primary miRNAs (pri-miRNAs), which are processed by the Drosha-DGCR8 complex into precursor miRNAs (pre-miRNAs). Pre-miRNAs are exported to the cytoplasm by Exportin-5 and cleaved by Dicer-TRBP into miRNA duplexes. The mature miRNA strand is loaded into the RNA-induced silencing complex (RISC), where Argonaute (AGO) proteins facilitate target mRNA degradation or translational repression through complementary base pairing.

miRNAs are small non-coding RNA molecules (≈22 nucleotides) that play a crucial role in post-transcriptional gene regulation, primarily by inhibiting protein translation or promoting mRNA cleavage (34). Their multi-step process of biogenesis involving both nuclear and cytoplasm. Initially, miRNA genes are transcribed by the RNA polymerase II (RNA poly II) into primary miRNA transcripts (pri-miRNAs), which are large, hairpin-structured RNA molecules capped with a 7-methylguanosine moiety and polyadenylated at the 3′ end. These pri-miRNAs are then recognized and processed in the nucleus by the microprocessor complex, comprising the RNase III enzyme Drosha and its cofactor DGCR8. The microprocessor complex cleaves the pri-miRNA near the base of its stem-loop structure to generate a ≈70-nucleotide precursor miRNA (pre-miRNA). The pre-miRNA is subsequently transported to the cytoplasm via Exportin-5 protein. In the cytoplasm, another RNase III enzyme Dicer, along with its partner TRBP (TAR RNA-binding protein), cleaves the pre-miRNA to produce a shorter RNA duplex. This duplex consists of the mature miRNA (guide strand) and its complementary strand (passenger strand). The mature strand is selectively incorporated into the RNA-induced silencing complex (RISC), where Argonaute (AGO) proteins serve as core components, while the complementary strand is typically degraded. Mature miRNAs within the RISC silence or regulate gene expression by binding to complementary sequences in target mRNAs. This interaction leads to gene silencing through two primary pathways: (1) target mRNA degradation, which involves perfect or near-perfect complementarity between the miRNA “seed region” (nucleotides 2–8) and the 3’UTR of target mRNAs. Upon incorporation into the RISC with AGO proteins, miRNAs guide the complex to these complementary sites, triggering endonucleolytic cleavage of the mRNA by AGO; (2) translation repression, when miRNA-mRNA binding is imperfect, the miRNA-RISC complex can suppress translation even with partial complementarity (35). Notably, miRNAs exhibit the capacity to regulate multiple target mRNAs concurrently, while a single mRNA may be subject to regulation by multiple miRNAs — dynamics that underscore the complexity of the miRNA-mRNA regulatory network (36) (**Figure 1**).

## 5 miRNA profiling tools

miRNA profiling has evolved into a sophisticated field with diverse methodologies tailored to detect and quantify miRNAs at different stages (e.g., primary, precursor, and mature miRNAs). The exploration of miRNA profiling tools in DR has provided critical insights into disease mechanisms and therapeutic targets. Numerous technologies have been utilized for miRNA detection and quantification including Northern blot, quantitative reverse transcriptase PCR (qRT-PCR), droplet-based digital PCR (ddPCR), miRNA microarrays, and other technologies. Below is an overview of each method, highlighting its strengths and limitations (**Table 1**).

**Table 1:** Different techniques for circulating miRNA quantification.

Technique	Advantages	Disadvantages	Key applications in DR research
Northern Blot	1. Widespread laboratory availability without specialized equipment. 2. Gold standard for validating novel miRNAs due to direct RNA detection.	1. Low sensitivity. 2. Time-consuming. 3. High RNA samples requirement.	Primarily used for miRNA verification in DR studies, though superseded by high-throughput methods in modern profiling.
qRT-PCR	1. Rapid and sensitive detection. 2. Cost-efficient and technically accessible for most labs. 3. Suitable for targeted miRNA quantification.	1. Limited throughput (handles one or few miRNAs per run). 2. Requires reference gene normalization for accuracy. 3. Relies on predefined sequences; cannot detect novel miRNAs. 4. LNA-modified primers may reduce amplification efficiency for low-abundance miRNAs.	Widely employed in DR research for focused miRNA quantification due to accessibility and speed.
ddPCR	1. Enables absolute quantification without reference genes or standard curves. 2. Exceptional sensitivity and precision. 3. Ideal for low-abundance miRNAs.	1. High cost due to specialized instrumentation. 2. Limited scalability for large-scale studies. 3. Practical challenges in routine DR biomarker discovery.	Used in DR studies requiring high precision, but high cost limits routine application.
miRNA Microarrays	1. Cost-efficient for high-throughput screening of large miRNA sets. 2. Suitable for broad profiling studies.	1. Requires substantial RNA input (similar to Northern Blot). 2. Lacks sensitivity for low-abundance miRNAs. 3.Confined to known miRNA sequences; no novel discovery. 4. Relative quantification only with narrow dynamic range.	Applied in DR for initial high-throughput screening to identify dysregulated miRNA patterns, but limitations in sensitivity hinder comprehensive analysis.

qRT-PCR, quantitative real-time polymerase chain reaction; ddPCR, droplet-based digital PCR; LNA, locked nucleic acid.

### 5.1 Northern blot

Northern blot, the first method for miRNA quantification following the initial discovery of lin-4 in 1993, remains a gold standard for validating novel miRNAs (37). It confirms RNA size via denaturing gel electrophoresis and miRNA-specific probes. Its advantages include widespread laboratory availability without specialized equipment. However, limitations include low sensitivity due to high RNA requirements (≥5-10 μg) and time-consuming procedures (2–3 days). This technique’s utility persists in miRNA verification despite being superseded by newer high-throughput methods in profiling studies (38).

### 5.2 Quantitative reverse transcriptase PCR

Compared to Northern blot, qRT-PCR offers rapid and sensitive detection of miRNAs, leveraging stem-loop primers or oligonucleotide-based approaches for reverse transcription and target-specific amplification, followed by quantification via sequence-specific probes. While stem-loop designs enhance primer binding specificity, locked nucleic acid (LNA)-modified primers further improve target recognition but may compromise amplification efficiency, posing challenges for quantifying low-abundance miRNAs. Although cost-efficient and technically accessible, qRT-PCR suffers from limited throughput, requires reference gene normalization for accurate relative quantification, and relies on predefined miRNA sequences—restricting discovery of novel miRNAs (39, 40).

### 5.3 Droplet-based digital PCR

ddPCR enables absolute miRNA quantification by partitioning reactions into thousands of nanoliter-scale droplets. This technique eliminates the need for standard curves or reference genes and achieves exceptional sensitivity and precision. However, its high cost, reliance on specialized instrumentation, and limited scalability for large-scale studies pose challenges for routine use in DR biomarker discovery (41).

### 5.4 miRNA microarrays

Fluorescently labeled miRNAs hybridize to array-immobilized probes, allowing simultaneous profiling of thousands of miRNAs. Though it is cost-efficient for high-throughput screening, microarrays require substantial RNA input, lack sensitivity for low-abundance miRNAs, and are confined to known sequences. Relative quantification and a narrow dynamic range further limit their utility in comprehensive DR studies (42).

## 6 Mechanistic roles of miRNAs in DR

**Figure 2:**
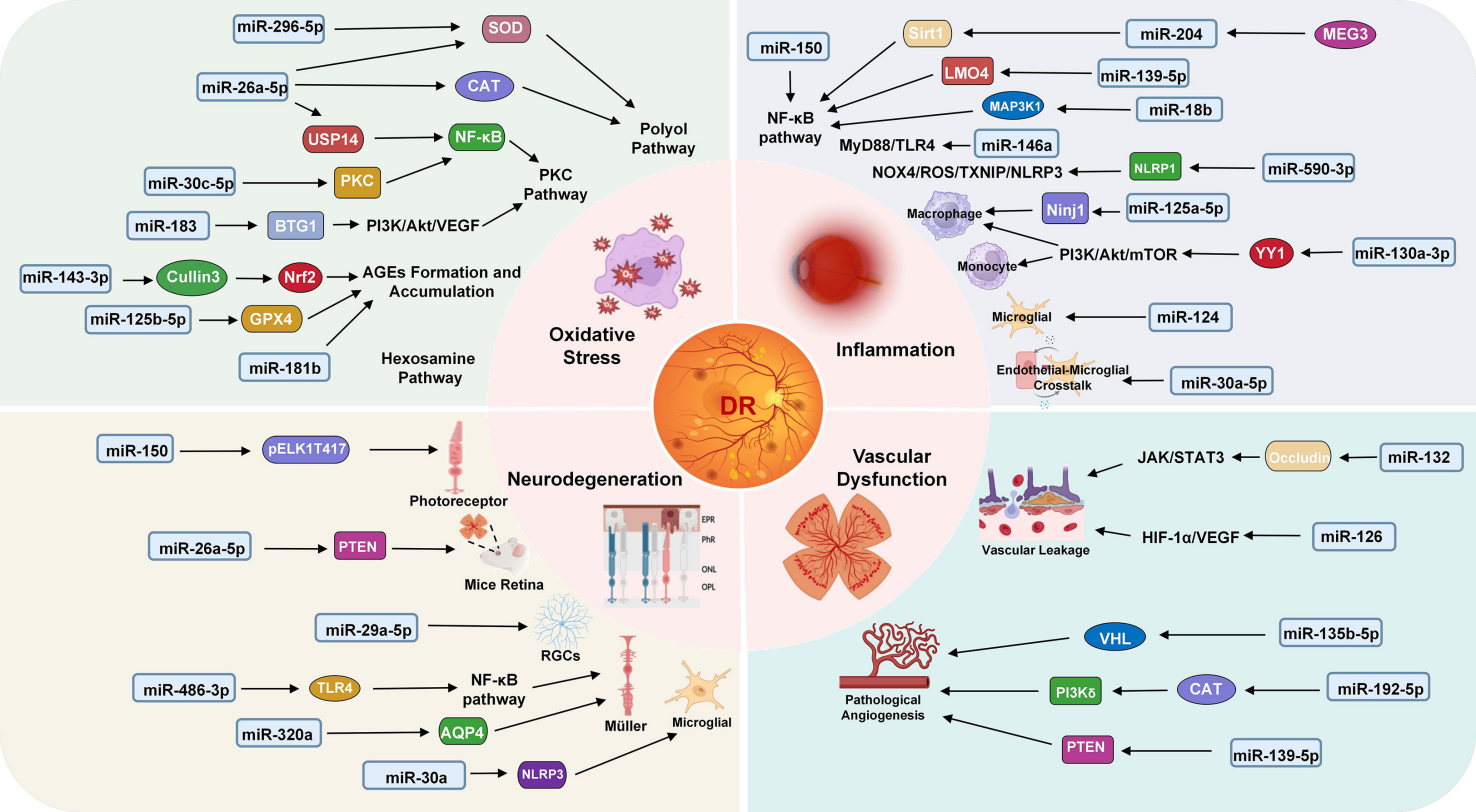
Mechanism of miRNAs in DR. Key miRNAs are involved in four major pathological processes: oxidative stress (miR-296-5p, miR-26a-5p, miR-30c-5p, miR183, miR-143-3p, miR-125b-5p, miR-181b), inflammation (miR-150, miR-204, miR-139-5p, miR-18b, miR-146a, miR-590-3p, miR-125a-5p, miR-130a-3p, miR-124, miR-30a-5p), neurodegeneration (miR-150, miR-26a-5p, miR-29a-5p, miR-486-3p, miR-320a, miR-30a) and vascular dysfunction (miR-132, miR-126, miR-135b-5p, miR-192-5p, miR-139-5p). Each miRNA modulates specific target genes and signaling pathways, contributing to DR progression.

miRNA biogenesis and silencing mechanisms are critical to post-transcriptional gene regulation. Dysregulation of these processes can exert profound impacts on various diseases, including systemic metabolic eye disorders such as DR. Within DR pathogenesis, miRNAs mediate key roles in modulating the major effects of DR progression (inflammation, oxidative stress, and vascular dysfunction), highlighting their involvement in orchestrating complex molecular pathways that underpin disease progression (43–45). Changes of miRNA levels in various tissues, organs, and blood have been reported across multiple studies in diabetic patients and animal models. In patients with T1DM, circulating miR-346, miR-148a, miR-181a, and miR-208 are upregulated, while miR-16, miR-93, miR-191, and miR-146a are downregulated (46, 47). Approximately 350 miRNAs are expressed, with at least 86 miRNAs dysregulated in the retinas of streptozotocin (STZ)-induced diabetic rats (48, 49). A growing focus lies in deciphering how miRNAs interact with target mRNAs to regulate cellular dysfunction, vascular permeability, neovascularization, and retinal neurodegeneration, thereby unraveling the underlying pathobiological mechanisms of DR (50). **Figure 2** illustrates the mechanistic roles of miRNAs in DR.

### 6.1 Oxidative stress

Oxidative stress, marked by pro-oxidants’ dominance over antioxidant defenses, drives cellular dysfunction via redox imbalance. While reactive oxygen species (ROS) normally regulate mitochondrial-lysosomal crosstalk during ATP production, excessive ROS accumulation damages biomolecules, exacerbates mitochondrial dysfunction, and causes uncontrolled oxidative tissue injury (51, 52). The core pathological mechanisms of DR are intimately linked to hyperglycemia-induced oxidative stress, with the polyol, hexosamine, protein kinase C (PKC) pathways, and formation of advanced glycation end products (AGEs) representing four central molecular networks (53–55). This section systematically discusses the action mechanisms of each pathway and research progress of miRNAs in DR oxidative stress.

#### 6.1.1 Activation of polyol pathway

Importantly, hyperglycemia activates the polyol pathway via aldose reductase (AR), leading to NADPH depletion and increased ROS (53, 56). The overexpression of miR-26a-5p attenuates DR by enhancing superoxide dismutase (SOD) and catalase (CAT) activities. In HG-exposed retinal Müller cells, miR-26a-5p overexpression elevated SOD/CAT levels, reduced oxidative markers (MDA, ROS), and inhibited mitochondrial cytochrome c release, alleviating oxidative stress. In STZ-induced diabetic male mice, miR-26a-5p agomir improved retinal histopathology and reduced oxidative markers (57). Another study found that miR-296-5p was downregulated in diabetic male mice retinal tissues, exacerbating DR progression. Restoring miR-296-5p alleviated DR pathology by targeting retinal ganglion cells: it reduced their apoptosis (altered Bcl-2/Bax/Caspase-3 expression) and decreased Evans blue leakage, indicating improved vascular integrity. Additionally, miR-296-5p upregulation suppressed oxidative stress (lowered VEGF and MDA, enhanced SOD activity) and inhibited GNAI2 expression by binding its 3’UTR (58).

#### 6.1.2 Activation of PKC pathway

PKC drives diabetic complications through hyperglycemia-induced diacylglycerol/ROS in retinal vasculature, triggering microvascular dysfunction via Nox, NF-κB, and VEGF (59, 60). Recent studies highlight miR-26a-5p as a critical regulator in DR pathogenesis. Specifically, *in vitro* experiments using HG-treated Müller cells exhibited downregulated miR-26a-5p, elevated oxidative stress, mitochondrial cytochrome c release, and NF-κB-driven inflammation (TNF-α, IL-1β, IL-6↑). Overexpression of miR-26a-5p mitigated these effects by directly targeting USP14, thereby suppressing USP14-mediated NF-κB activation (p-IκBα, p-p65↓) and nuclear translocation. Extending these findings to *in vivo* models, STZ-induced diabetic male mice displayed similar miR-26a-5p downregulation, retinal oxidative injury, and inflammatory responses, which were ameliorated by miR-26a-5p agomir treatment (57). The overexpression of miR-183 in DR rats activates the PI3K/Akt/VEGF signaling pathway by directly targeting and suppressing BTG1, thereby promoting vascular endothelial cell proliferation, pathological angiogenesis (via upregulated CD34 and eNOS), and ROS accumulation. Conversely, miR-183 silencing upregulated BTG1, inhibiting oxidative damage and pro-angiogenic pathways (61). Furthermore, miR-30c-5p acts as a critical regulator of DR-associated vascular dysfunction by suppressing PLCG1, thereby inhibiting the PKC/NF-κB pathway in HG-treated human retinal endothelial cells (HRECs) (62).

#### 6.1.3 Formation and accumulation of AGEs

AGEs disrupt extracellular matrix (ECM) integrity and bind to RAGE to activate the NADPH oxidase/NF-κB/MAPK pathways, thereby exacerbating DR (53). Studies have demonstrated that AGEs-stimulated Müller cells exhibited elevated ROS and inflammation, which were mitigated by miR-143-3p-mediated inhibition of Cullin3 neddylation, thereby stabilizing Nrf2 to enhance antioxidant responses (63). Under AGEs-induced HRECs, miR-125b-5p suppresses P53-mediated ferroptosis by restoring glutathione peroxidase 4 (GPX4) activity and blocking lipid peroxidation, thereby preserving blood-retina barrier (BRB) integrity (64). Beyond retinal cells, systemic investigations in STZ-induced diabetic male mice revealed miR-181b downregulation exacerbated endothelial dysfunction by amplifying oxidative stress and vascular inflammation. AGEs suppressed miR-181b expression in human renal arteries and human umbilical vein endothelial cells, correlating with elevated superoxide production and impaired vasodilation. miR-181b mimics reversed these effects, reducing oxidative stress and restoring endothelial function. These findings suggest that miR-181b may mitigate oxidative stress in vascular endothelial cells, highlighting its therapeutic potential for DR (65).

#### 6.1.4 Activation of hexosamine pathway

Hyperglycemia activates the hexosamine pathway via glutamine: fructose-6-phosphate amidotransferase (GFAT), producing UDP-N-acetylglucosamine (UDP-GlcNAc) that drives pathological O-glycosylation (66). O-GlcNAc modifications drive pathogenesis by activating AMPK and inducing photoreceptor degeneration in retinal neurons (67). To date, no studies have reported that miRNAs are involved in DR via activation of the hexosamine pathway; this still warrants further investigation.

### 6.2 Inflammation and immune cell activation

Hyperglycemia directly promotes inflammatory gene expression and activates immune cells via multiple pathways, amplifying inflammatory mediator production and impairing vascular endothelial cell structural and functional integrity (68). miRNAs further exert multidimensional regulatory roles in DR pathology by targeting inflammatory mediators, signaling pathways, and immune cell functions (69, 70). This section systematically discusses its mechanisms from three dimensions: inflammatory signaling pathway modulation, immune cell recruitment and activation, and miRNA-endothelial-immune cell crosstalk.

#### 6.2.1 Inflammatory signaling pathway modulation

The dysregulation of inflammatory pathways in DR is centrally orchestrated by miRNAs through their targeted modulation of the NF-κB pathway, a master regulator of pro-inflammatory cytokine production (e.g., TNF-α, IL-1β, IL-6). miR-150, an intrinsic anti-inflammatory regulator, suppresses NF-κB activation under physiological conditions; however, its diabetes-associated depletion exacerbates ocular inflammation by disinhibiting NF-κB-driven responses, as evidenced by LPS-induced endothelial cell models (71–73). Furthermore, the MEG3/miR-204/Sirt1 axis mitigates inflammation via NF-κB pathway that disrupts pro-inflammatory cytokine production (e.g., TNF-α, IL-6) (74). Similarly, miR-18b suppresses MAP3K1-dependent phosphorylation of NF-κB p65, reducing vascular leakage and retinal thickening in STZ-induced diabetic SD rats, while miR-139-5p targets LMO4 to block NF-κB activation and downstream inflammatory cascades (TNF-α, IL-6, Cox-2) in HG-incubated human retinal pigment epithelial (ARPE-19) cells (75, 76). Apart from NF-κB, miR-146a attenuates inflammation in HG-treated primary human retinal microvascular endothelial cells (hRMECs) by inhibition of MyD88/TLR4 signaling and suppression of TNF-α production, positioning it as a pivotal regulator of retinal endothelial dysfunction (77). Additionally, miR-590-3p plays an active role in inflammation by directly targeting NLRP1, leading to the inhibition of pyroptosis via the NOX4/ROS/TXNIP/NLRP3 signaling cascade (78, 79). Collectively, the dysregulation of inflammatory signaling pathways in DR is centrally orchestrated by miRNAs through their targeted modulation of key transcriptional regulators and downstream effectors.

#### 6.2.2 Immune cell recruitment and activation

The regulation of immune cells dynamics in DR involves miRNA-mediated modulation of chemokine signaling, cellular adhesion, and inflammatory cascades. miR-125a-5p directly targets Ninj1, a key mediator of macrophage adhesion and pro-inflammatory factor release. By suppressing Ninj1, miR-125a-5p reduces macrophage infiltration into inflamed retinas and attenuates vascular leakage in both endotoxin-induced and STZ-induced diabetic mice models, underscoring its role in preserving vascular integrity (80). Concurrently, overexpression of miR-130a-3p attenuated DR progression by targeting YY1 to inhibit the PI3K/Akt/mTOR pathway, thereby promoting macrophage autophagy, reducing M1 polarization, and suppressing inflammation in HG-treated human monocyte (THP-1) and STZ-induced diabetic male mice (81). miR-124 further modulates immune homeostasis by normalizing HG-induced microglial hyperactivity, suppressing inflammatory mediators (e.g., Tnf-α, Ccl2, Ccl3), and downregulating transcription factors (PU.1) and lipid raft proteins (Flot1), thereby preventing vasoregression and neuroretinal dysfunction (48).

#### 6.2.3 miRNA-endothelial-immune cell crosstalk

In DR, miRNA-mediated regulation of intercellular adhesion molecule-1 (ICAM-1) and endothelial-immune crosstalk critically drives inflammatory pathology. Studies reveal elevated ICAM-1 expression in retinal vessels, promoting leukocyte adhesion, vascular leakage, and endothelial injury (82). miR-146a directly modulates this pathway by targeting IRAK1 and ICAM-1, with its rhythmic expression in diabetic STZ-induced diabetic rat retinas inversely correlating with ICAM-1 oscillation, suggesting circadian regulation of vascular inflammation (83). Apart from ICAM-1, miR-30a-5p emerges as a dual regulator of endothelial-microglial crosstalk. In a mice model of ischemic retinopathy, miR-30a-5p inhibition reduced pathological neovascularization by enhancing FasL+ microglial interactions with Fas+ endothelial cells, promoting endothelial apoptosis and microglial phagocytosis (84).

### 6.3 Retinal neurodegeneration

Emerging evidence reveals that neurodegeneration in DR, characterized by neuronal dysfunction, apoptosis, and reactive gliosis, precedes microvascular changes (85). Hyperglycemia-induced metabolic stress triggers tau hyperphosphorylation and mitochondrial dysfunction in retinal ganglion cells (RGCs), while oxidative stress and neuroinflammation reduce neurotrophic factors (86, 87). Activated microglia and Müller cells exacerbate neuronal damage through cytokine release and glutamate disruption (88–90).

Previous studies have shown that miRNAs critically regulate the neurodegenerative mechanisms (91). Specifically, in T2D mice, deletion of miR-150 (miR-150^-^/^-^) exacerbates photoreceptor apoptosis detected by increased TUNEL staining in the retina. This effect occurs because decreased miR-150 promotes nuclear pELK1T417 translocation, identified as the key step triggering photoreceptor apoptosis in response to the diabetic/high-fat conditions (92). Similarly, miR-26a-5p is downregulated in STZ-induced diabetic mice retina, exacerbating neuronal apoptosis. Mechanistically, miR-26a-5p directly targets PTEN, suppressing its upregulation in inner/outer nuclear layers and dampening glial activation (via GFAP reduction) and inflammatory markers (IL-1β, NF-κB, VEGF) (93). Transitioning to human studies, miR-29a-5p levels were markedly elevated in DR patients’ blood, correlating with hyperglycemia and dyslipidemia. HG upregulates miR-29a-5p expression in RGCs. This upregulation induced RGCs apoptosis, oxidative stress (increased ROS/MDA, decreased SOD), and inflammation (elevated TNF-α/IL-6) via SIRT3 suppression (94). Moreover, in HG-treated Müller cells, miR-486-3p overexpression reduced oxidative stress, inflammation, and apoptosis by targeting TLR4 to repress NF-κB signaling (95). Similarly, miR-320a overexpression reduced hypoxia-induced damage of Müller cells by suppressing aquaporin-4 (AQP4) expression, inhibiting superoxide anion production, enhancing cell viability, and promoting AQP4 internalization, thereby alleviating retinal edema. Conversely, miR-30a activates microglia in an NLRP3-dependent manner, thus promoting the progress of DR (96).

### 6.4 Vascular dysfunction

Pathological angiogenesis and vascular hyperpermeability are hallmark features of DR, driven by dysregulated miRNAs that modulate endothelial proliferation, junctional integrity, and angiogenic signaling cascades. In angiogenesis, miR-139-5p emerges as a critical promoter of retinal neovascularization, where its upregulation in HG-exposed RMECs enhances VEGF production, cell migration, and tube formation by repressing PTEN, a key suppressor of the PI3K/Akt pathway (97). Similarly, miR-135b-5p promotes endothelial cell proliferation and angiogenesis by inhibiting Von Hipp-el-Lindau (VHL) expression, as demonstrated through *in vitro* retinal endothelial cell isolation from DR mice transfected with miR-135b-5p inhibitor or VHL-overexpressing plasmids, and *in vivo* using a STZ-induced diabetic mice model (98). Conversely, miR-192-5p acts as a protective regulator by suppressing ELAVL1-mediated PI3Kδ stabilization, thereby inhibiting endothelial proliferation and migration in HG-treated hRMECs, with its overexpression mitigating pathological angiogenesis (99).

Interestingly, in the field of vascular permeability and stability, miR-132 disrupts retinal barrier integrity by directly targeting occludin, a tight junction protein, via JAK/STAT3 activation in HG-stressed ARPE-19 cells, exacerbating vascular leakage and epithelial mobility. Pharmacological inhibition of miR-132 restores occludin/E-cadherin expression and reduces permeability (100). Meanwhile, miR-126 downregulation in diabetic retinas plays a pivotal role in vascular destabilization. Extracellular vesicles (EVs) from mesenchymal stem cells under diabetic-like conditions suppress miR-126 in retinal pericytes, amplifying HIF-1α/VEGF-driven pericyte loss and BRB dysfunction. miR-126 deficiency correlates with enhanced vascular leakage, independent of Ang-2/PDGF pathways, positioning miR-126 restoration as a strategy to counteract EV-mediated BRB breakdown (101).

## 7 Circulating miRNAs: DR biomarkers

miRNAs, identified as highly stable molecules in various biological fluids, can be efficiently extracted from blood and other liquid biopsies (102). Emerging evidence demonstrates that dynamic alterations in miRNA expression profiles reflect pathological progression across diverse diseases, including cancers, cardiovascular diseases, and even DR (103, 104). For instance, miR-29a-3p exhibits stage-specific dysregulation during colorectal cancer progression, correlating with tumor invasiveness and metastatic potential (105). Similarly, miR-21 has emerged as a pivotal regulator in cardiovascular pathologies, driving cardiac remodeling (106). Notably, while the functional roles of these miRNAs in retinal tissues require further elucidation, their diagnostic utility is underscored by large-scale studies. Circulating miRNAs in biofluids (e.g., plasma, serum, aqueous humor, vitreous humor) not only reflect pathological states but also hold represent promising non-invasive biomarkers for early diagnosis and therapeutic monitoring (107). **Table 2** summarizes the literature review regarding the expression profiles of miRNAs in DR.

**Table 2:** Potential diagnostic miRNA biomarkers for DR.

miRNA	DR stage	Dysregulation	Sample type	Detection method	Ref.
miR-335-3p	—	down	plasma	qRT-PCR	(108)
miR-26a-5p	NPDR	down	plasma	qRT-PCR	(109)
miR-4448, miR-338-3p, miR-485-5p, miR-9-5p	NPDR	down	serum	RNA-seq	(110)
miR-190a-5p	NPDR	up	serum	RNA-seq	(110)
miR-93	NPDR, PDR	down	serum	qRT-PCR	(111)
miR-152	NPDR, PDR	up	serum	qRT-PCR	(111)
miR-146a, miR-21	NPDR, PDR	up	serum	qPCR	(112)
miR-34a	NPDR, PDR	down	serum	qPCR	(112)
hsa-miR-24-3p, hsa-miR-197-3p, hsa-miR-3184-3p	PDR	up	vitreous humor	RT-qPCR	(113)
hsa-miR-20a-5p, hsa-miR-23b-3p, hsa-miR-142-3p, hsa-miR-185-5p, hsa-miR-223-3p, hsa-miR-362-5p, hsa-miR-662	PDR	up	vitreous humor	RT-qPCR	(114)
miR-199a-5p, hsa-miR-326, miR-100-5p	PDR	down	vitreous humor	RT-qPCR	(115)
miR-126	PDR	down	vitreous humor	qPCR	(116)
miR-185-5p, miR-17-5p, miR-20a-5p, miR-15b-5p, miR-15a-5p	DME	down	aqueous humor	RT-qPCR	(117)
let-7b, miR-320b, miR-762, miR-4488	NPDR	up	vitreous humor	microarrays	(118)
miR-455-3p, miR-296	PDR	up	aqueous humor	microarrays	(118)

NPDR, non-proliferative DR; PDR, proliferative DR; DME, diabetic macular edema; qRT-PCR, quantitative real-time polymerase chain reaction; qPCR, quantitative PCR; RT-qPCR, reverse transcription-quantitative PCR; RNA-seq, RNA sequencing.

Some studies have shown that blood-derived miRNAs possess remarkable predictive value for the diagnosis of DR. Through qRT-PCR analysis, plasma miR-335-3p levels were significantly decreased in DR patients and demonstrated specificity in distinguishing DR cases from healthy individuals and T2DM patients, suggesting its utility as a non-invasive biomarker for DR screening (108). Similarly, qRT-PCR quantification showed markedly reduced plasma miR-26a-5p levels in T2DM patients with NPDR compared to those without retinopathy, correlating with superior retinal nerve fiber layer (RNFL) thickness (109). RNA-seq profiling of serum identified five differentially expressed miRNAs (miR-4448, miR-338-3p, miR-485-5p, miR-9-5p, and miR-190a-5p) in NPDR patients, with the first four downregulated and miR-190a-5p upregulated (110). Furthermore, qRT-PCR analysis revealed dynamic expression patterns of miR-93 and miR-152 during diabetes progression in serum: miR-93 levels decreased (OR=0.25, p = 0.028), while miR-152 levels increased (OR=1.37, p < 0.001) across diabetes, NPDR, and PDR cohorts (111). Notably, qPCR assays revealed serum miR-146a and miR-21 levels correlating with DR severity (PDR > severe NPDR > moderate > mild > normal fundus), while miR-34a is in the opposite tendency (112).

The available data on the expression profiles of miRNAs in the vitreous humor and aqueous humor of eyes with DR remain scarce, primarily due to ethical considerations that preclude the use of healthy individuals as controls. Using RT-qPCR, studies with non-diabetic controls (macular hole cases) identified upregulated miRNAs in vitreous analyses of PDR patients: hsa-miR-24-3p, hsa-miR-197-3p, hsa-miR-3184-3p correlated with VEGF-A/TGF-β levels, while hsa-miR-20a-5p, hsa-miR-23b-3p, hsa-miR-142-3p, hsa-miR-185-5p, hsa-miR-223-3p, hsa-miR-362-5p, and hsa-miR-662 showed elevation compared to controls (113, 114). Conversely, miR-199a-5p and hsa-miR-326 were downregulated in PDR vitreous, and miR-100-5p demonstrated diagnostic potential through its reduction (114, 115). qPCR analysis stratifying PDR severity stages (IV-VI) against idiopathic macular hole controls revealed progressive miR-126 downregulation correlating with advanced fibrovascular proliferation in vitreous (116). In contrast, aqueous humor studies employed different control cohorts: RT-qPCR comparisons between diabetic macular edema (DMO) patients and cataract controls identified five markedly downregulated miRNAs (miR-185-5p, miR-17-5p, miR-20a-5p, miR-15b-5p, miR-15a-5p) (117). A comprehensive pilot study employing miRNA 3.0 microarrays systematically profiled DR subtypes (T1DM with PDR, T2DM with PDR, T2DM with NPDR) against non-diabetic vitreoretinal surgery controls, revealing fluid-specific patterns: vitreous showed predominant miRNA upregulation (e.g., let-7b, miR-320b, miR-762, miR-4488), while aqueous exhibited subtype-unique markers (miR-455-3p in T2DM with NPDR; miR-296 in T2DM with PDR) (118). Though limited by heterogeneous control groups (macular pathologies, cataracts), these findings collectively suggest specific miRNA signatures reflecting DR progression, with vitreous and aqueous humor profiles changes potentially serving as accessible proxies for intraocular pathophysiology.

## 8 miRNA-targeted retinal therapies

**Figure 3:**
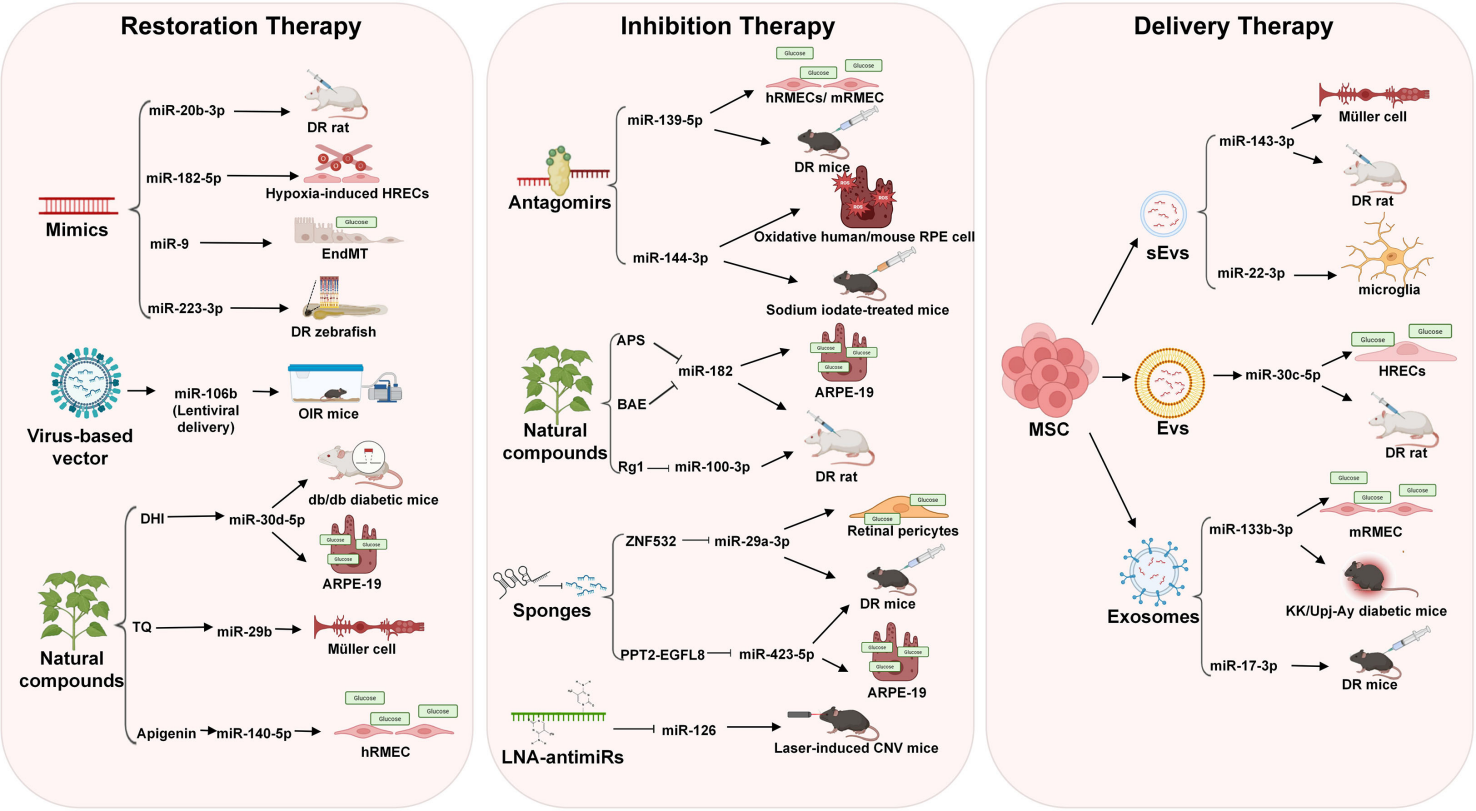
Major strategies for miRNA-targeted therapy in DR. Three main approaches include: miRNA restoration therapy (including miRNA mimics, natural compounds, and virus-based vectors), miRNA inhibition therapy (including miRNA antagomirs, natural compounds, miRNA sponges, and LNA-antimiRs), and miRNA delivery therapy (including mesenchymal stem cell (MSC)-derived extracellular vesicles (EVs), small extracellular vesicles (sEVs), and exosomes).

Emerging understanding of miRNAs in biology and their dysregulation in many diseases has prompted scientists to investigate their potential use in DR. Current strategies focus on three principal modalities: (1) miRNA restoration therapy, which introduces synthetic oligonucleotides to supplement downregulated or non-functional miRNAs; (2) miRNA inhibition therapy, which employs antagonists to suppress overexpressed miRNAs; (3) miRNA delivery therapy, where advances in systems like extracellular vesicles and exosomes enhance therapeutic precision by improving miRNA bioavailability and minimizing off-target effects. **Figure 3** illustrates miRNA-targeted therapeutic strategies in DR.

### 8.1 miRNA restoration therapy

miRNA mimics are double-stranded RNA oligonucleotides that exactly copy the mature miRNA duplex to replenish downregulated miRNAs (119). Studies found that miR-20b-3p mimics attenuated retinal inflammation in DR rat models by targeting TXNIP, leading to reduced inflammatory cytokine levels and improved retinal pathology (120). In HRECs, miR-182-5p mimics suppressed hypoxia-induced angiogenesis by downregulating ANG and BDNF, thereby enhancing cell viability and stabilizing vascular integrity (121). Furthermore, miR-9 mimics counteracted glucose-induced endothelial-to-mesenchymal transition (EndMT) by inhibiting TGF-β and proinflammatory pathways, effectively reducing retinal vascular leakage (122). Interestingly, miR-223-3p mimics in zebrafish models exhibited dual effects: while elevating glucose levels, they simultaneously mitigated retinal vascular degeneration and restored structural abnormalities in ganglion and nuclear layers through VEGF-A/NRP-1 pathway modulation (123). Furthermore, a miRNA-encoding plasmid or virus-based vector could be exploited for purposes requiring miRNA delivery for a specific period. In the oxygen-induced retinopathy (OIR) mice model, lentiviral delivery of miR-106b reduced pathological neovascularization by approximately 45-50%, achieving a comparable therapeutic efficacy to anti-VEGF treatment, which underscores the therapy potential in counteracting pathological angiogenesis in PDR (124).

While miRNA mimics hold therapeutic potential for DR, their clinical application is hampered by side-effects linked to the passenger strand activity, raising concerns over safety and specificity (125). In contrast, natural compounds derived from traditional medicine offer a promising alternative by restoring endogenous miRNA expression. Danhong injection (DHI) alleviates DR by upregulating miR-30d-5p to suppress JAK1 expression, as demonstrated in both db/db diabetic mice and HG-induced ARPE-19 cells, through suppressing inflammation, improving renal/retinal injury, and inhibiting pathological angiogenesis (126). Similarly, thymoquinone (TQ), a bioactive phytochemical, alleviates Müller cell apoptosis in diabetic mice by elevating miR-29b levels, thereby repressing SP1-mediated activation of pro-apoptotic factors (e.g., Bax and Caspase-3) and enhancing anti-apoptotic Bcl-2 expression (127). Furthermore, Apigenin attenuates DR by elevating miR-140-5p expression to inhibit the HDAC3/PTEN/PI3K/AKT pathway, as demonstrated in HG-induced hRMECs through reduced angiogenesis, proliferation, and migration *in vitro* (128). These findings highlight the capacity of herbal constituents to fine-tune miRNA networks without exogenous oligonucleotide delivery, thereby preserving retinal homeostasis through multi-target mechanisms while minimizing side effects.

### 8.2 miRNA inhibition therapy

miRNA inhibition therapy represents a strategic approach to counteract disease progression by selectively suppressing the activity of aberrant miRNAs within target tissues, as demonstrated in various pathologies. For example, the synergistic downregulation of miR-92a and miR-18a in non-small cell lung cancer inhibits EMT and suppresses tumor advancement through SPRY4 targeting (129). In diabetic complications like nephropathy, the downregulation of miR-21 enhances PPARα expression to improve mitochondrial function and alleviate lipid metabolism disorders, thereby mitigating disease progression — a mechanism highly relevant to targeting similar pathways in DR (130). This therapy is achievable through diverse methodologies such as miRNA antagomirs, natural compounds, miRNA sponges, and LNA-antimiRs, which effectively disrupt miRNA-mRNA interactions (102).

Antagomirs achieve miRNA suppression through sequence-specific degradation of target miRNAs by utilizing chemically engineered, cholesterol-conjugated antisense oligonucleotides that bind complementarily to miRNAs (131). For example, the miR-139-5p antagomir ameliorates DR by suppressing miR-139-5p expression to upregulate PTEN and inhibit VEGF-driven retinal neovascularization, as demonstrated in HG-treated hRMECs/mice retinal microvascular endothelial cells (mRMECs) through reduced migration, tube formation, and VEGF levels, and further validated in diabetic mice models by attenuating acellular capillaries and pathological blood vessel formation (97). Similarly, The miR-144-3p antagomir mitigates oxidative stress-induced retinal degeneration in DR by suppressing miR-144-3p to activate Nrf2-dependent antioxidant signaling (e.g., NQO1, GCLC), demonstrated in oxidatively stressed human/mice RPE cells (reduced apoptosis) and sodium iodate-treated mouse (preserved retinal integrity via subretinal delivery) (132).

Emerging evidence highlights natural compounds as promising miRNA-modulating agents for DR management. Astragalus polysaccharide (APS) mitigates mitochondrial apoptosis in ARPE-19 cells by suppressing the miR-182/Bcl-2 axis under high glucose conditions. APS downregulates miR-182, restoring Bcl-2 expression, reducing cytochrome-c release, and attenuating apoptosis markers (Bax, cleaved caspases) (133). Similarly, blueberry anthocyanins (BAE) alleviate DR via the miR-182/OGG1 axis, suppressing HG-induced ROS, endoplasmic reticulum stress (ERS), and apoptosis in ARPE-19 cells. BAE inhibits miR-182 to upregulate OGG1, confirmed by luciferase assays, and ameliorates retinal oxidative damage in DR rats (134). Complementarily, ginsenoside Rg1 targets the miR-100-3p/FBXW7/c-MYC axis, inhibiting angiogenesis in hRMECs and DR rat models. Rg1 downregulates miR-100-3p, elevating FBXW7 to promote c-MYC degradation, thereby reducing vascular leakage and pathological neovascularization (135). These studies collectively demonstrate that natural compounds modulate distinct miRNA pathways to address multifactorial DR pathogenesis.

miRNA sponges represent a class of artificially engineered RNA molecules meticulously designed to specifically inhibit the function of one or more target miRNAs, thereby serving as potent tools in miRNA inhibition therapy; these constructs typically comprise long RNA chains embedded with multiple (ranging from 4 to 10 or more) tandem, fully or nearly fully complementary binding sites that exhibit high affinity for the designated miRNAs, functioning analogously to a “sponge” to efficiently sequester and bind the target miRNAs within cellular environments through competitive interactions (136, 137)—crucially, this sequestration prevents the miRNAs from binding to their natural downstream mRNA targets, effectively nullifying their gene silencing capabilities and restoring normal gene expression (138). For instance, circular RNA ZNF532 acts as a miR-29a-3p sponge to sequester and reduce miR-29a-3p activity, thereby upregulating NG2, LOXL2, and CDK2 expression to rescue pericyte function in HG-exposed retinal pericytes and STZ-induced diabetic mice, highlighting its relevance in mitigating early DR complications (139). Similarly, in the context of PDR, studies reveal that the long noncoding RNA PPT2-EGFL8 functions as a sponge for miR-423-5p. In PDR, long noncoding RNA PPT2-EGFL8 functions as a miR-423-5p sponge, suppressing hypoxia-induced hRMECs proliferation and ameliorating pathological neovascularization in STZ-induced diabetic mice models by modulating PPARD/ANGPTL4 signaling, thereby illustrating the targeted efficacy of miRNA sponges in addressing advanced DR pathologies (140).

LNA-antimiRs, characterized by their 20-O and 40-C methylene-bridged ribose ring modifications that confer structural rigidity, exhibit enhanced nuclease resistance and superior miRNA binding affinity compared to antagomirs (141). The study by Zhou et al. demonstrated that LNA-anti-miRs for miR-126 effectively suppressed pathological choroidal neovascularization (CNV) in a laser-induced mice model and reduced VEGF-A production through αB-Crystallin promoter regulation in RPE cells (142). Notably, while this study validates LNA-antimiRs as potent tools for modulating ocular angiogenesis, direct evidence for LNA-mediated miRNA inhibition in DR remains unexplored.

### 8.3 miRNA delivery therapy

Current miRNA delivery approaches include conjugation (direct ligand-conjugated miRNAs enabling targeted delivery but inducing hepatic aggregation), viral vectors (efficacy limited by safety concerns), and nanoparticles (cationic carriers protecting miRNAs but risking cytotoxicity) (143–145).

Emerging non-viral strategies, such as EVs and exosomes, offer promising alternatives due to their biocompatibility and targeted delivery potential for DR treatment (146). MSC-sEVs demonstrated therapeutic efficacy by delivering miR-143-3p to suppress NEDD8-mediated Cullin3 neddylation, thereby stabilizing Nrf2 to reduce oxidative stress and inflammation in Müller cells - both *in vitro* (restoring AGEs-induced endothelial barrier dysfunction) and in diabetic mice (alleviating retinal inflammation and gliosis) (63). Similarly, MSC-sEVs attenuated DR progression in diabetic rats through miR-22-3p transfer, which inhibited NLRP3 inflammasome activation in microglia, subsequently reducing retinal inflammation and blood-retinal barrier damage (147). Human umbilical cord mesenchymal stem cell-derived extracellular vesicles (MSC-Evs) were shown to enrich miR-30c-5p, targeting PLCG1 to suppress PKC/NF-κB signaling pathways, thereby ameliorating inflammatory responses in both STZ-induced diabetic rats and HG-treated HRECs (62). Exosomes, as a specialized subset of EVs, have demonstrated enhanced targeting capabilities in DR models. Bone marrow MSC-derived exosomes delivered miR-133b-3p to mRMECs, effectively suppressing FBN1-mediated angiogenesis and oxidative stress while promoting apoptosis in hyperglycemic conditions, as evidenced by both *in vitro* cellular assays and KK/Upj-Ay diabetic mice models (148). Similarly, umbilical cord MSC exosomes enriched with miR-17-3p demonstrated therapeutic efficacy by modulating the STAT1/miR-17-3p/VEGF axis to suppress pathological neovascularization in DR mice (149). These findings position EV/exosome-mediated miRNA delivery as a transformative approach for DR treatment, combining the physiological benefits of natural biomaterials with precise molecular targeting capabilities to address multifactorial retinal pathophysiology. A summary of the current miRNA delivery platforms is provided in **Table 3**.

**Table 3:** Comparison of miRNA delivery platforms for DR.

Delivery platform	Mechanism	Advantages	Limitations
Conjugation	Direct ligand-conjugated miRNAs	Targeted delivery	Hepatic aggregation, limited tissue specificity
Viral Vectors	Recombinant viruses encoding miRNA mimics or inhibitors	High transfection efficiency, persistent expression	Immunogenicity
Nanoparticles	Cationic lipids encapsulating miRNAs	Protects miRNA	Cytotoxicity, limited retinal penetration
Extracellular Vesicles	Natural membrane vesicles carrying miRNAs	Biocompatible, low immunogenicity	Scalable production challenges

## 9 Limitations and translational challenges

There are several critical limitations that impede clinical translation of miRNA-based therapies. Firstly, the heavy reliance on animal models—such as STZ-induced diabetic rodents—poses inherent constraints. These models do not fully recapitulate the complex, multifactorial, and chronic nature of human DR, particularly in terms of metabolic comorbidities, genetic diversity, and the slow progression of microvascular and neuroglial pathology (150). Species-specific differences in retinal anatomy, miRNA expression profiles, and disease progression limit the direct applicability of these findings to humans. Secondly, while circulating miRNAs show promise as non-invasive biomarkers, their clinical utility is hampered by preanalytical variability (e.g., sample collection, processing, and storage), lack of standardized profiling methods, and heterogeneity in expression profiles across different patient populations and stages of DR (151, 152). Moreover, current miRNA therapeutic strategies face substantial hurdles in clinical implementation. Key challenges include ensuring efficient, targeted, and sustained delivery to retinal tissues while minimizing off-target effects and immune activation (119). Current delivery systems—such as viral vectors, lipid nanoparticles, and extracellular vesicles—still face limitations in bioavailability, tissue specificity, and potential cytotoxicity (153).Furthermore, the risk of off-target effects due to the pleiotropic nature of miRNAs, which can regulate multiple mRNA targets, raises concerns about unintended consequences on physiological processes (154). Long-term safety profiles, including immunogenicity and cumulative toxicity, remain largely unexplored.

## 10 Discussion and future directions

While the compelling evidence underscores the immense potential of miRNAs as sensitive diagnostic biomarkers for non-invasive liquid biopsy-based staging and multi-target therapeutic agents capable of modulating key hyperglycemia-induced pathways (oxidative stress, inflammation, angiogenesis, neurodegeneration) in DR, significant translational challenges necessitate focused future research, including rigorously validating the diagnostic accuracy, specificity, and predictive power of candidate miRNAs across diverse, large-scale longitudinal cohorts and ethnic populations using standardized, sensitive profiling techniques like ddPCR to overcome limitations of variability and establish clinically actionable thresholds; developing efficient, targeted, and stable delivery systems capable of safely and persistently delivering miRNA mimics or inhibitors specifically to retinal cells while minimizing off-target effects and immune responses; conducting comprehensive preclinical studies evaluating long-term efficacy, safety, and potential synergies or antagonisms with existing therapies (e.g., anti-VEGF, faricimab); advancing robust miRNA-based combinatorial strategies that integrate multi-miRNA therapeutics or miRNA modulation with emerging modalities like sustained-release devices or gene editing technologies to enhance durability and efficacy; and fostering the integration of validated circulating miRNA signatures with cutting-edge diagnostic tools like AI-driven retinal imaging and metabolomics into unified, accessible platforms for precision risk stratification, early detection, personalized intervention, and real-time monitoring of DR progression to ultimately shift the paradigm towards prevention and halt the relentless global burden of this vision-threatening complication.
